# A potential role of Fgf3 for epibranchial formation in zebrafish

**DOI:** 10.3389/fcell.2025.1652723

**Published:** 2025-08-20

**Authors:** Haewon Jeon, Sil Jin, Saehoon Joo, Yucheol Choe, Min Young Lee, Chong Pyo Choe

**Affiliations:** ^1^ Division of Applied Life Science, Gyeongsang National University, Jinju, Republic of Korea; ^2^ Division of Life Science, Gyeongsang National University, Jinju, Republic of Korea; ^3^ Plant Molecular Biology and Biotechnology Research Center, Gyeongsang National University, Jinju, Republic of Korea

**Keywords:** epibranchial, branchial basket, pharyngeal arches, Fgf3, zebrafish

## Abstract

In fish, epibranchials are dorsal facial skeletal elements composing the branchial basket, which articulate with ceratobranchials ventrally and pharyngobranchials dorsally. They form in the posterior pharyngeal arches through endochondral bone formation. In zebrafish, the development of epibranchial structures has not been described in detail at the genetic and cellular levels compared to the development of the jaw skeleton. Here, we report the developmental process of epibranchial formation in zebrafish and the genetic requirement of Fgf3 in this process. In contrast to the simultaneous formation of epibranchial cartilages 1-4 reported in other fish, we observe a sequential development of epibranchial cartilages posteriorly to anteriorly in zebrafish. While in other fish, epibranchial cartilages develop independently from the ceratobranchial cartilages as a separate chondrification center, we show that their formation is associated with the dorsal end of ceratobranchial cartilages. This occurs as chondrocytes bud out from the dorsal end of ceratobranchial cartilages. Finally, we suggest that Fgf3 is necessary to develop epibranchial cartilages, possibly by controlling the proliferation of chondrocytes at the dorsal end of ceratobranchial cartilages. Our results provide a novel insight into the development of epibranchials and establish a genetic and cellular basis to investigate their developmental mechanism.

## Introduction

In two crown groups of living gnathostomes, Osteichthyes (bony fishes) and Chondrichthyes (cartilaginous fishes, including sharks, rays, and chimaeras), the branchial basket plays an important role in food chewing, processing, and transport between the buccal cavity and the esophagus (Vandewalle et al., 2000). Generally, the branchial basket is composed of a series of articulated rods, with unpaired median basibranchials, followed by paired hypobranchials, ceratobranchials, epibranchials, and pharyngobranchials, ventrally to dorsally (Pradel et al., 2014). During embryogenesis, they arise in the posterior pharyngeal arches, with the Meckel’s and palatoquadrate cartilages developing in the first pharyngeal arch and the ceratohyal and hyosymplectic cartilages in the second pharyngeal arch (Schilling and Kimmel, 1997; Gillis et al., 2009). The ventral ceratobranchial (Cb) cartilages are serially homologous to the Meckel’s and ceratohyal cartilages, and the dorsal epibranchial (Eb) cartilages are serially homologous to the palatoquadrate and hyosymplectic cartilages (Schilling and Kimmel, 1997). The Eb cartilage articulates with the corresponding Cb cartilage ventrally and the pharyngobranchial (Pb) cartilage dorsally (Carvalho et al., 2013) and may develop flanges for attachment of the dorsal gill muscles as well as support dermal plates of the pharyngeal teeth (Carvalho et al., 2013). Eb5, associated with the last pharyngeal arch, is not found in Chondrichthyes and most Osteichthyes, with smaller Eb5 than Ebs 1-4 being found in actinopterygians (Gillis et al., 2009; Carvalho et al., 2013). However, developmental studies on Eb5 of actinopterygians show that it originates from the Cb4 in pharyngeal arch 6. It is not considered homologous to Ebs and is renamed as the accessory element of the ceratobranchial 4 (AECb4) (Carvalho et al., 2013).

Development of Eb has been described in various fish and follows the typical steps of endochondral bone formation: mesenchymal condensations, chondrification, growth, and ossification (Kronenberg, 2003). In three species of actinopterygians, *Prochilodus argenteus*, *Lophiosilurus alexandri*, and *Pseudoplatystoma corruscans*, when the branchial basket begins to form, with all Cb cartilages already formed and being stained, Eb cartilages 1-4 first become visible at the dorsal end of the corresponding Cb cartilages, although they have not yet been stained (Carvalho et al., 2013). The appearance of all 4 Ebs, either simultaneously or within a short time interval early in development, has also been described in other ostariophysan species (Tewari, 1971; Engeman et al., 2009; Block and Mabee, 2012). Then, Eb cartilages 1-4 chondrify at the dorso-lateral end of the corresponding Cb cartilages, with Eb cartilage 4 being the largest and the other elements progressively smaller anteriorly (Carvalho et al., 2013). Soon after, when the branchial basket forms completely, AECb4 appears at the dorso-lateral end of Cb4, close to Eb4 (Carvalho et al., 2013). Later, Eb cartilages 1–4 develop uncinate processes that are pointed anterodorsally, with AECb4 remaining a slender cartilaginous bar that almost extends to the uncinate process of Eb4 (Carvalho et al., 2013). Finally, Eb cartilages 1–4 ossify at the middle of the cartilage, extending towards both ends, with AECb4 never ossify (Carvalho et al., 2013). Eb cartilages arise in a separate mesenchymal condensation from that of corresponding Cb cartilages, whereas AECb4 arises in the same chondroblastic layer (Carvalho et al., 2013). Similarly, in the Little Skate, *Leucoraja erinacea*, Eb cartilages 1-5 originate as an independent condensation in the corresponding pharyngeal arches 3-7 at 42 mm total length (TL), followed by chondrification at 50 mm TL, then articulate ventrally with the corresponding Cb cartilages 1–5 (Gillis et al., 2009).

In zebrafish, the development of Ebs was also described along with other skeletal elements arising in pharyngeal arches 3–7. Formation and ossification of the ventral Cb cartilages precede those of the dorsal Eb cartilages (Cubbage and Mabee, 1996; Schilling and Kimmel, 1997). Eb cartilages 1-4 arise at the dorsal end of respective Cb cartilages 1-4, with the ossification co-occurring at 6.4 mm standard length (SL) and progressing sequentially posterior to anterior at approximately 7.2–8.0 mm SL (Cubbage and Mabee, 1996). On the posterior edge of Ebs 1-4, uncinate processes forms (Cubbage and Mabee, 1996). Ebs 1-4 grow, and the ventral and dorsal ends of Ebs 1-4 articulate with the corresponding Cbs and the Pbs (Cubbage and Mabee, 1996). Consistently, endochondral growth zones are identified at the ventral and dorsal ends of Ebs 1-4 in 13.5 mm SL zebrafish (Heubel et al., 2021; Le Pabic et al., 2022). When Eb cartilages ossify, Eb cartilage 5 appears separately posterior to Eb4 and anterior to the dorsal end of Cb cartilage 5 (Cubbage and Mabee, 1996). However, the early development of Ebs, such as mesenchymal condensations and specification of chondrocytes, has yet to be described, and genetic and cellular analysis is absent in Eb development.

Here, we report the early developmental processes of Ebs in zebrafish. In contrast to the near-simultaneous appearance of Eb cartilages in other fish, we report a sequential emergence of Eb cartilages posteriorly to anteriorly in zebrafish. Although it has been known that Eb cartilages arise as an independent condensation from Cb cartilages in other fish, we show that their formation is associated with the dorsal end of Cb cartilages in zebrafish. Specifically, they occur at the dorsal end of Cb cartilages through the budding of chondrocytes. Furthermore, we propose that Fgf3 is necessary to develop Eb cartilages, probably by controlling the proliferation of chondrocytes at the dorsal end of Cb cartilages.

## Materials and methods

### Zebrafish lines

All zebrafish (*Danio rerio*) were handled as described previously (Kimmel et al., 1995). All zebrafish work was approved by Gyeongsang National University Institutional Animal Care and Use Committee. To follow the development of epibranchials, 30 embryos were grown as a batch in a 28.5°C incubator; for 3 dpf, they were reared in 100 mL of embryonic medium (5.03 mM NaCl. 0.17 mM KCl. 0.33 mM CaCl_2_⋅ 2H_2_O. 0.33 mM MgSO_4_⋅ 7H_2_O. 0.1% (w/v) Methylene blue) and then were grown in 2000 mL of fish-system water until subsequent analysis. SL was measured as described previously (Parichy et al., 2009; Owen and Kelsh, 2021). *fgf10* ^*tbvbo*^, *pax1a* ^*GNU25*^, *Tg(sox10:EGFP)* ^*ba2*^, and *Tg(nkx2.3:Gal4VP16)* ^*el93*^ lines as well as *Tg(UAS:DN-Fgfr1)* and *Tg(UAS:DN-EphB4a)* transgenic constructs, were published (Fischer et al., 2003; Herzog et al., 2004; Carney et al., 2006; Choe et al., 2013; Choe and Crump, 2015; Jin and Choe, 2024; Jeon et al., 2025). *Tg(hsp70I:DN-Fgfr1)* ^*GNU115*^ line was generated using the Gateway (Invitrogen) Tol2kit (Kwan et al., 2007). *fgf3* ^*GNU48*^ mutant was generated with the CRISPR/Cas9 system (Hwang et al., 2013). 150 pg of gRNA and 150 pg of mRNA encoding a nuclear-localized Cas9 were injected into one-cell stage wild-type Tübingen (TU) embryos. The injected embryos were grown and then outcrossed to wild-type TU animals to identify zebrafish bearing in/del mutations in the *fgf3* gene. One *fgf3* mutant line (*fgf3* ^*GNU48*^) was secured. For genotyping of *fgf3* ^*GNU48*^, PCR amplicons produced with primers *fgf3*_GT_F and *fgf3*_GT_R were digested with Tsp45I, with a wild-type fragment producing 216 and 136 bp and mutant fragment generating 347 bp. See Supplementary Material and Methods for *fgf3* gRNA oligo and genotyping primers as well as for the characterization of *fgf3* ^*GNU48*^ mutants.

### Quantitative real-time PCR

Total RNA was isolated from the wild type and *fgf3* mutant at 48 hpf using the NucleoSpin RNA Plus kit (MACHEREY-NAGEL). Approximately 30 embryos were used for each RNA preparation. 1 μg of total RNA was utilized to synthesize cDNA using ReverTra Ace™ High Efficient Reverse Transcriptase (TOYOBO). Quantitative real-time PCR (qRT-PCR) was conducted as described previously (Jeon et al., 2022). GraphPad Prism was applied for statistical analysis, with data analyzed using an unpaired t-test, with significance determined at p < 0.05.

### Measurement of viability and standard length in *fgf3* mutants

Viability and standard length were measured together in each fish from 4 dpf. For analysis, individual fish was handled in a Petri dish (90 mm × 15 mm). Each fish was examined for survival at 10 a.m. every day. To reduce stress due to anesthesia, the standard length of each fish was measured every other day at 2 PM. *fgf3* mutants and their wild-type siblings carrying *Tg(sox17:GFP)* transgene were identified in real-time at 36 hpf based on the defects in pharyngeal pouches for analysis (Herzog et al., 2004; Li et al., 2019) and then were genotyped right after death. GraphPad Prism was applied for survival and growth plots.

### Staining

Alcian blue and alizarin red staining, fluorescent double *in situ* hybridizations, and BrdU and Lysotracker staining were performed as described (Zuniga et al., 2011). Riboprobe to *fgf3* was published (Choe and Crump, 2014). For riboprobes to *hand2*, *jag1b*, *dlx2a*, *dlx3b*, *dlx4a*, *barx1*, and *sox9a*, PCR amplicons were cloned into pGEM®-T Easy Vector Systems (Promega), linearized, and then digoxigenin- or dinitrophenol-labeled RNAs were synthesized using T7 or SP6 RNA polymerase (Roche). See Supplementary Material and Methods for primers.

### Imaging

Craniofacial cartilages and bones were dissected manually with fine insect pins, flat-mounted, and photographed on an Olympus BX50 upright microscope using mosaic V2.1 software. Fluorescent images were captured with an Olympus FV3000 confocal microscope using FV31S-SW software. After capturing approximately 80 μm Z-stacks at 3.0 and 1.5 μm intervals with Olympus UPLXAPO ×20 and ×40 objective lenses, maximum intensity projections encompassing static confocal sections were assembled using FV31S-SW software. Any adjustments were applied to all panels using Adobe Photoshop.

## Results

### Epibranchial cartilages appear sequentially from posterior to anterior

**FIGURE 1:**
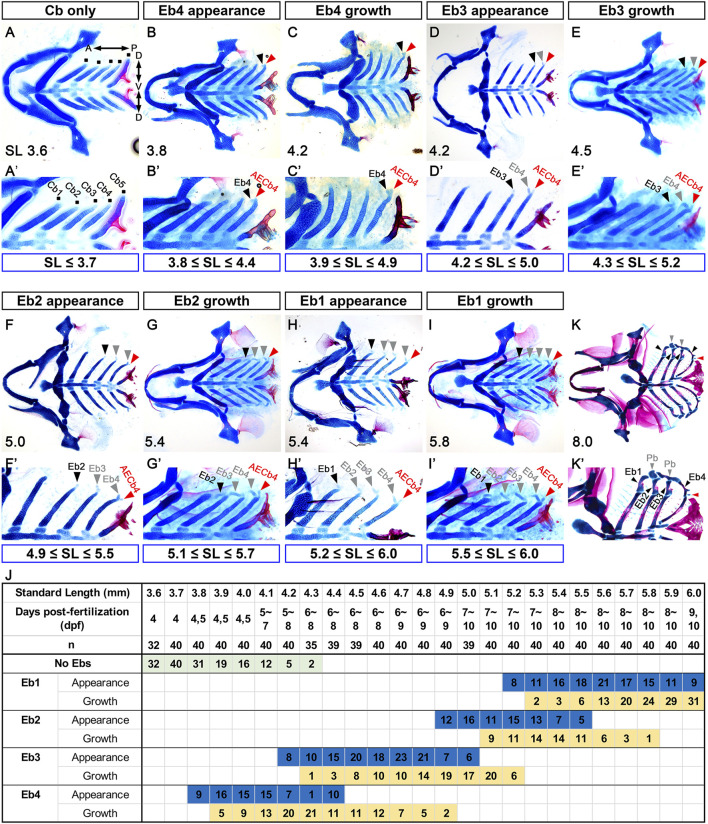
Sequential formation of epibranchial cartilages posteriorly to anteriorly. **(A–I)** Ventral views of dissected facial skeletal elements stained with Alcian Blue (cartilage) and Alizarin Red (bone). **(A’–I’)** Cropped images of one side of the corresponding A-I. The range of SL at each stage of Eb development is marked below. **(A,A’)** Five pairs of Cbs are seen, with the dorsal ends of five Cbs on one side marked with dots. **(B,B’)** A tiny piece of Eb4 at the dorsal end of the fourth Cb is indicated by black arrowheads, with the posterior dorsal tip of the fourth Cb marked with red arrowheads. **(C,C’)** Black arrowheads indicate growing Eb4. AECb4 is evident at the dorsal end of the fourth Cb and marked with red arrowheads. **(D,D’)** The emerging Eb3 at the dorsal end of the third Cb is marked with black arrowheads, with the Eb4 and AECb4 indicated by grey and red arrowheads, respectively. **(E,E’)** Black arrowheads mark growing Eb3, with grey and red arrowheads indicating Eb4 and AECb4, respectively. **(F,F’)** Eb2 appearing at the dorsal end of the second Cb is indicated by black arrowheads, along with Ebs 3 and 4 marked with grey arrowheads. Red arrowheads indicate AECb4. **(G,G’)** Black arrowheads indicate growing Eb2, with grey and red arrowheads indicating Ebs 3 and 4 and AECb4, respectively. **(H,H’)** Eb1 at the dorsal end of the first Cb is marked with black arrowheads, with Ebs 2-4 and AECb4 indicated by grey and red arrowheads, respectively. **(I,I’)** Black arrowheads mark growing Eb1, with growing Ebs 2-4 and AECb4 indicated by grey and red arrowheads. **(J)** Timeline of the development of the epibranchial cartilages. Dpf indicates the number of days post-fertilization to reach the corresponding SL, with “n” indicating the number of fish examined at each SL. The number in the colored box is the number of fish in the same SL at the corresponding developmental stages. AECb4 formation that seems to coincide with Eb4 formation is not scored. **(K,K’)** Ventral views of dissected facial skeletal elements stained with Alcian Blue (cartilage) and Alizarin Red (bone) at 8 mm SL. Black arrowheads indicate 4 Ebs, with red and grey arrowheads indicating AECb4 and 2 Pbs, respectively. Each facial skeletal element is labeled. **(K’)** Cropped image of one side of **(K)**. Anterior is to the left. Dorsal is at the top in **(A’–K’)**. Cb, ceratobranchial cartilage; Eb, epibranchial cartilage; AECb4, accessory element of the ceratobranchial 4; Pb, pharyngobranchial cartilage; SL, standard length; A, anterior; P, posterior; D, dorsal; V, ventral.

To follow the development of Ebs, we visualized facial cartilages and bones with alcian blue and alizarin red staining in 3.6–6.0 mm SL zebrafish. Before describing Eb development, we should note that the developmental states of Eb varied significantly among individual fish, even at the same SL, depending on the days post-fertilization (dpf) required to reach that SL (Figure 1J). In 3.6 mm SL zebrafish, five pairs of Cbs were established at 4 dpf before Ebs appeared (Figures 1A,A’). Although Ebs were not seen up to 4.3 mm SL in some zebrafish, the first sign of Eb cartilage was seen at 3.8–4.4 mm SL, in which a tiny anteriorly pointed cartilage appeared at the dorsal end of Cb4 (black arrowheads in Figures 1B,B’; Figure 1J). The tiny cartilage was Eb4 and grew anterior-dorsally (black arrowheads in Figures 1C,C’; Figure 1J). Coincidentally, as Eb4 was growing, a tiny piece of cartilage was evident posterior to Eb4 at the dorsal end of Cb4 (red arrowheads in Figures 1C,C’), which was AECb4 as described in actinopterygians (Carvalho et al., 2013). AECb4 seemed to appear at 3.8 mm SL, together with Eb4 (red arrowheads in Figures 1B,B’). In 4.2–5.0 mm SL zebrafish, a small piece of Eb3 directed anteriorly appeared at the dorsal end of Cb3 and grew anterior-dorsally (black arrowheads in Figures 1D,D’,E,E’; Figure 1J). In 4.9–5.5 mm SL zebrafish, Eb2 appeared at the dorsal end of Cb2, which was also directed anteriorly, and grew anterior-dorsally (black arrowheads in Figures 1F,F’,G,G’; Figure 1J). Eb1 appeared lastly at the dorsal end of Cb1 in 5.2–6.0 mm SL zebrafish and grew anterior-dorsally (black arrowheads in Figures 1H,H’,I,I’; Figure 1J). Once all Ebs formed, they continued to grow and change shape, and then ossification progressed in the middle regions of each Eb at 8.0 mm SL (black arrowheads in Figures 1K,K’), as described previously (Cubbage and Mabee, 1996). While Ebs developed, AECb4 was maintained as a tiny piece of cartilage at the dorsal end of Cb4 (red arrowheads in Figures 1B–K’). The sequential appearance of Eb cartilages described here differs from the emergence of all 4 Ebs, either simultaneously or within a short interval reported in other fish (Tewari, 1971; Engeman et al., 2009; Block and Mabee, 2012). It may reflect a derived developmental mode of zebrafish Eb formation. Alternatively, it is feasible that a series of populations of alcian blue-negative chondrocytes would form at the dorsal end of the corresponding Cb cartilages either simultaneously or within a short time interval like other fish but then would become alcian blue-positive cartilage cells sequentially from Eb 4 to 1.

### Chondrocytes for epibranchial cartilages are unlikely to arise from separate chondrification centers from those for ceratobranchial cartilages

Given that the dorsal palatoquadrate and hyosymplectic cartilages and the ventral Meckel’s and ceratohyal cartilages arise from separate skeletogenic precursors in the first and second pharyngeal arches (Mork and Crump, 2015; Paudel et al., 2022), it would be possible that the dorsal Eb and ventral Cb cartilages arise from separate skeletogenic precursors along the dorso-ventral axis in pharyngeal arches 3–7. We investigated this possibility using molecular markers for dorso-ventral patterning, skeletogenic mesenchymal condensations, and chondrocytes in pharyngeal arches (Thomas et al., 1997; Talbot et al., 2010; Alexander et al., 2014; Barske et al., 2016). At 36 and 42 hpf, *hand2* and *jag1b* were expressed in the respective ventral and dorsal domains of all pharyngeal arches 3-7, even though the segmental expression of *jag1b* in the dorsal regions of pharyngeal arches 4-7 was somewhat unclear (Supplementary Figures S1A,B). At 36 hpf, the expression of *dlx4a* and *dlx3b* marking the intermediate domain of the first and second arches was seen in arches 3-6 and 3–5 (Supplementary Figures S1C,E). At 42 hpf, *dlx4a* and *dlx3b* were expressed in the intermediate domain of arches 3-6, but they were not expressed in arch 7, the last arch (Supplementary Figures S1D,F). Thus, like the first and second arches, all arches 3-7 seemed to be patterned into the dorsal and ventral domains at 36 hpf, with the intermediate domain appearing further patterned by 42 hpf in all but the last arches.

**FIGURE 2:**
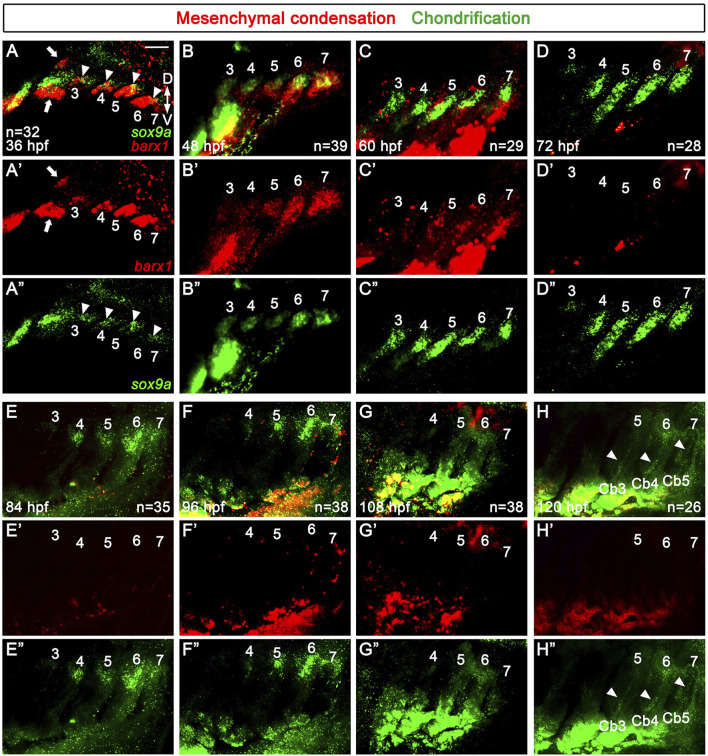
Specifications of skeletogenic mesenchyme and chondrocytes in the posterior pharyngeal arches. **(A–H)** Fluorescence *in situ* hybridization of *barx1* (red) and *sox9a* (green) at 36 to 120 hpf. Expression domains of *barx1* and *sox9a* mark respective skeletogenic mesenchyme and chondrocytes in pharyngeal arches 3 to 7. **(A)** At 36 hpf, a single domain expressing *barx1* is observed in all pharyngeal arches 3 to 7, with two separate populations (arrows) seen in the dorsal and ventral regions of the second arch. *sox9a* is partially expressed in a subdomain of the *barx1*-expressing domain in pharyngal arches 3 to 6 (arrowheads). **(B)** At 48 hpf, *barx1* expression in more anterior pharyngeal arches 3 and 4 gradually reduces, with a single domain of *sox9a* expression evident in all posterior arches 3–7. **(C)** At 60hpf, a single domain expressing *sox9a* is maintained in each posterior arch, with the shape of *sox9a* expressing domains becoming elongated. *barx1* expression domains are seen in the adjacent regions to the *sox9a*-expressing domains in the posterior arches 3–7. **(D)** At 72 hpf, the single domain of *sox9a* expression is further elongated in pharyngeal arches 4-7, with *sox9a* expression in arch 3 faded. **(E)** At 84 hpf, *sox9a* expression is seen in the dorsal regions of arches 4-7, with its expression almost abolished in arch 3. **(F)** At 96 hpf, *sox9a* expression is observed in the dorsal regions of arches 4-7, but its expression fades in arch 4. **(G)** Traces of *sox9a* expression remain in the dorsal area of arches 4-6 at 108 hpf. **(H)** At 120 hpf, traces of *sox9a* expression are seen in the dorsal area of arch 6, with its expression barely seen in the dorsal region of arch 5 and abolished in arch 4. Arrowheads indicate *sox9a* expression in putative Cbs 3–5. **(D–H)** From 72 to 120 hpf, *barx1* expression is no longer observed in the posterior arches 3–7. **(E–H)** Expression of *barx1* and *sox9a* in the developing gills is seen in the lower area of **(E–H). (A’–H’)** Red channel only. **(A”–H”)** Green channel only. Scale bar: 40 μm. Anterior is to the left. Dorsal is at the top. Cb, ceratobranchial cartilage; D, dorsal; V, ventral. n, number of animals analyzed.

At 36 hpf, the skeletogenic mesenchymal condensations occurred as a single population in all arches 3-7, as evidenced by a single domain expressing *barx1* in each posterior arch (Figures 2A,A’). This was in contrast to the two separate populations of *barx1*-positive mesenchyme in the dorsal and ventral areas of the second arch (arrows in Figures 2A,A’). At 36 hpf, *sox9a* expression was seen in a subpopulation of the *barx1*-expressing skeletogenic mesenchyme in arches 3–6 (arrowheads in Figures 2A,A”). At 48 hpf, *sox9a* expression was also observed in arch 7 (Figures 2B,B”), with *barx1* expression being gradually decreased from anterior to posterior (Figures 2B,B’). Consequently, a single population of *sox9a*-expressing chondrocytes was specified in each of the posterior arches by 48 hpf. At 60 hpf, the expression of *sox9a* persisted in each posterior arch, with its domains elongating along the dorso-ventral axis (Figures 2C,C”). The expression of *barx1* was maintained in the neighboring regions of the *sox9a*-expressing domains in each posterior arch (Figures 2C,C’). At 72 hpf, the single domain expressing *sox9a* had further elongated, although its expression in arch 3 had faded (Figures 2D,D”). Likely, the *sox9a*-expressing chondrocytes within the elongated domain of each posterior arch contributed to the formation of Cb cartilages between 60 and 72 hpf. From 72 to 120 hpf, the expression of *barx1* was barely seen in arches 3–7 (Figures 2D–H, D′–H′). Compared to its expression at 72 hpf, *sox9a* expression at 84 hpf was faded and restricted to the dorsal area of arches 4-7, with its expression in arch 3 almost abolished (Figures 2E,E”). At 96 hpf, when alcian blue-positive Cb cartilage formation is completed, *sox9a* expression was seen in the dorsal area of arches 4-7, with its expression in arch 4 faded (Figures 2F,F”). While Cb cartilages were forming in the posterior arches from 36 to 96 hpf, no additional and separate *barx1*-expressing skeletogenic mesenchymal condensations or *sox9a*-expressing chondrification centers for Eb cartilages were observed in the dorsal areas of respective posterior arches.

At 108 hpf, which corresponds to about 3.6–3.9 mm SL, traces of *sox9a* expression were observed in the dorsal area of arches 4-7, with its expression barely seen in arch 7 (Figures 2G,G”). At 120 hpf, which corresponds to about 3.8–4.2 mm SL, when the bud of Eb4 is just beginning to appear in arch 6, traces of *sox9a* expression were still seen in the dorsal area of arch 6, but its expression was nearly abolished in the dorsal area of arch 5 (Figures 2H,H”). Notably, weak expression domains of *sox9a* reappeared in the putative Cbs 3 to 5 at 120 hpf (arrowheads in Figures 2H,H”). Nevertheless, no additional or separate domains expressing *barx1* or *sox9a* reemerged in the dorsal area of the posterior arches, apart from the initial domains of *barx1*-expressing skeletogenic mesenchymal condensations and *sox9a*-expressing chondrification centers from 36 to 120 hpf. This result suggests that the chondrocytes for the dorsal Eb cartilages are unlikely to form from independent chondrification centers, unlike those for the ventral Cb cartilages. This contrasts with the distinct mesenchymal condensations and independent chondrification centers observed in the dorsal and ventral areas of the first and second arches during the development of the dorsal palatoquadrate and hyosymplectic cartilages and the ventral Meckel’s and ceratohyal cartilages (Paudel et al., 2022).

### Epibranchial cartilage 4 forms by budding from the dorsal end of ceratobranchial cartilage 4

**FIGURE 3:**
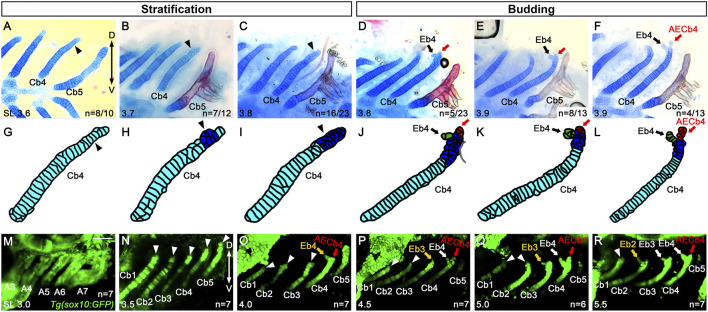
Budding of epibranchial cartilage 4 at the dorsal end of ceratobranchial cartilage 4. **(A–F)** Alcian Blue (cartilage) and Alizarin Red (bone) staining visualizing Cbs. **(A)** Arrowhead indicates rectangular and mono-layered Alcian Blue-positive cartilage cells in the dorsal end of Cb4. 8 out of 10 animals show mono-layered cartilage cells, whereas 2 out of 10 animals show multi-layered cartilage cells presented in **(B). (B)** A population of round and multi-layered cartilage cells in the dorsal end of Cb4 is marked with an arrowhead. 7 out of 12 animals show multi-layered cartilage cells, whereas 5 out of 12 animals show mono-layered cartilage cells. **(C)** Arrowhead indicates the extended population of round and multi-layered cartilage cells to the dorsal end of Cb4. 16 out of 23 animals show multi-layered cartilage cells, whereas 2 out of 23 animals show mono-layered cartilage cells. The remaining 5 out of the 23 animals form the Eb4 bud presented in **(D). (D)** Arrow marks the Eb4 budding anterior-dorsally at the dorsal end of Cb4, with a red arrow marking the posterior dorsal tip. 5 out of 23 animals display the Eb4 bud, with the remaining 18 out of the 23 animals showing mono- or multi-layered cartilage cells but no Eb4 bud, as presented in **(A–C)**. **(E)** Arrow indicates the Eb4 bud, with increasing cartilage cells in the Eb4 bud, at the dorsal end of Cb4. Red arrow marks the posterior dorsal tip of Cb4. 8 out of 13 animals show an increase in the Eb4 bud, whereas 1 out of 13 animals shows multi-layered cartilage cells, yet no Eb4 bud formation. The remaining 4 out of the 13 animals show a growing Eb4 presented in **(F). (F)** The growing Eb4 and the evident AECb4 at the dorsal end of Cb4 are marked with black and red arrows, respectively. **(G–L)** Cartoons of Cb4 forming Eb4 drawn based on the corresponding **(A–F)**. **(M–R)** Confocal images of Cbs in *Tg(sox10:GFP)* transgenic animals during Eb formation. **(M)** *sox10*-positive cells label the posterior pharyngeal arches (A3 to A7) in 3.0 mm SL zebrafish. **(N)** Arrowheads indicate the dorsal ends of Cbs 1-5 in 3.5 mm SL zebrafish. Before Eb4 forms at the dorsal end of Cb4, no distinct populations of *sox10*-positive cells are observed around the dorsal regions of Cbs 1–5. **(O–R)** Yellow and white arrows mark Ebs, with arrowheads indicating the dorsal ends of corresponding Cbs in 4.0 **(O)** 4.5 **(P)** 5.0 **(Q)** and 5.5 mm SL zebrafish **(R)**. No separate populations of *sox10*-positive cells are seen around the dorsal regions of respective Cbs before Eb formation. AECb4 is marked with red arrows. Scale bar: 40 μm. Anterior is to the left. Dorsal is at the top. A, arch; Cb, ceratobranchial cartilage; Eb, epibranchial cartilage; AECb4, accessory element of the ceratobranchial 4; D, dorsal; V, ventral. n, number of animals analyzed.

To better understand the emergence of Eb, we analyzed Eb4 formation in 3.6–3.9 mm SL zebrafish at the cellular level. In 3.6 mm SL zebrafish, in which Eb4 has yet to form, most cells in the dorsal region of Cb4 were rectangular and mono-layered (arrowheads in Figures 3A,G). In 3.7 mm SL zebrafish, a population of the rectangular and mono-layered cells in the dorsal region transitioned into rounded and multi-layered cells (arrowheads in Figures 3B,H), which was extended to the dorsal end of Cb4 in 3.8 mm SL zebrafish (arrowheads in Figures 3C,I). In 3.8 mm SL zebrafish, within the population of rounded and multi-layered cells at the dorsal end of Cb4, some cells did bud out anteriorly, the first emergence of Eb4 (black arrows in Figures 3D,J). The cells in the bud increased and rearranged to grow Eb4 in 3.9 mm SL zebrafish (black arrows in Figures 3E,F,K,L). During the budding of Eb4, cells remaining at the very dorsal end of Cb4 seemed to become AECb4 continuously, which was evident when the bud of Eb4 began to grow (red arrows in Figures 3D–F,J–L). The stratification of cartilage cells, followed by budding of Eb4 at the dorsal end of Cb cartilage, differs from the previously described emergence of Eb cartilage as a separate center of chondrification in other fish (Carvalho et al., 2013).

To verify further whether Eb cartilages originated from chondrocytes at the dorsal end of Cb cartilages or arose independently as a separate chondrification center, we followed cells contributing to Eb cartilages with *Tg(sox10:GFP)* animals that label ectomesenchyme in the pharyngeal arches contributing to facial cartilages (Liu et al., 2023). In 3.0 mm SL zebrafish, *sox10*-positive cells comprised the posterior pharyngeal arches 3 to 7 (Figure 3M). In 3.5 mm SL zebrafish, *sox10*-positive cells contributed to chondrocytes of Cb cartilages 1 to 5, but no separate populations of *sox10*-positive cells were seen in the adjacent regions to the dorsal end of the corresponding Cb cartilages (arrowheads in Figure 3N). In 4.0 mm SL zebrafish, *sox10*-positive cells formed a bud of Eb4 at the dorsal end of Cb4 (yellow arrow in Figure 3O), but separate *sox10*-positive populations from *sox10*-positive Cbs 1 to 3 were not still observed (arrowheads in Figure 3O). In 4.5 and 5.0 mm SL zebrafish, when a bud of Eb3 seemed to appear (yellow arrow in Figure 3P) or was evident (yellow arrow in Figure 3Q) at the dorsal end of Cb3, no *sox10*-positive populations that were distinguished from those of Cbs were seen around the dorsal end of Cbs 1 and 2 (arrowheads in Figures 3P,Q). In 5.5 mm SL zebrafish, it was similar in that when a bud of Eb2 appeared (yellow arrow in Figure 3R), distinguishable *sox10*-positive cell populations were not observed in the neighboring area around the dorsal region of Cb1 (arrowhead in Figure 3R). Although we could not follow further *sox10*-positive Cbs in 6.0 mm or longer SL zebrafish due to technical issues, this result supports that the epibranchial buds originate not in an independent chondrification center but within the dorsal end of the corresponding Cb cartilages.

### Epibranchial cartilage formation is associated with the dorsal end of ceratobranchial cartilage

**FIGURE 4:**
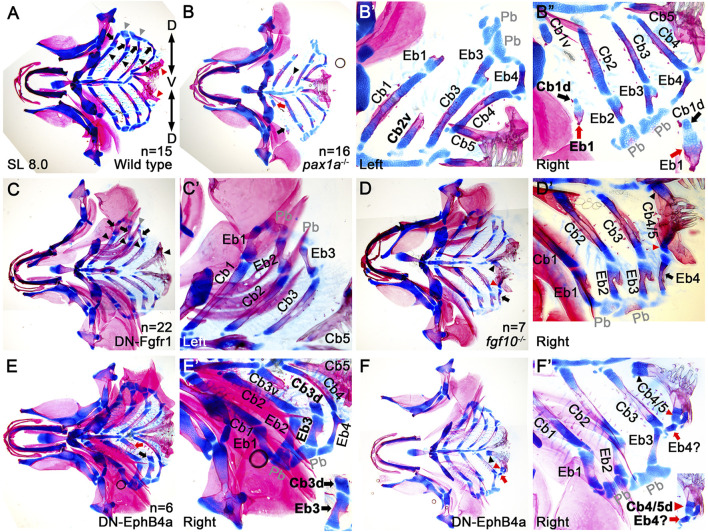
Association of the dorsal end of ceratobranchial cartilage with epibranchial cartilage formation. **(A–F)** Ventral views of dissected facial skeletal elements stained with Alcian Blue (cartilage) and Alizarin Red (bone) at 8.0 mm SL. Each facial skeletal element is labeled. Anterior is to the left. **(A)** In wild types, black and red arrowheads, arrows, and grey arrowheads indicate five Cbs, 4 Ebs, and 2 Pbs on one side. Red arrowheads mark the dorsal ends of the fifth Cbs, which completely ossify (red), with no Alcian Blue-positive cartilage (blue) present. **(B)** In a *pax1a* mutant, a hypoplastic Cb2 that loses the dorsal region is marked with an arrowhead. A dorsal piece of the hypoplastic Cb1, with the corresponding Eb, is indicated by a black arrow. Red arrow marks a ventral piece of the hypoplastic Cb1. **(B’)** A high-magnification image of the left side of the *pax1a* mutant in **(B)**. Dorsal is at the top. **(B”)** A high-magnification image of the right side of the *pax1a* mutant in **(B)**, with a cropped image of the dorsal piece of hypoplastic Cb1 and the corresponding Eb1 in the lower right corner. Dorsal is at the bottom. **(C)** Black arrowheads, arrows, and grey arrowheads indicate four Cbs, 3 Ebs, and 2 Pbs on the left side of the compound animals transiently expressing a dominant negative form of Fgfr1 receptor (DN-Fgfr1) in the *nkx2.3*-positive pharyngeal endoderm. On the left side, Cb4 and the corresponding Eb4 are missing. On the right side, five Cbs and 4 Ebs form normally. **(C’)** A high-magnification image of the left side of the compound animal in **(C)**. Dorsal is at the top. **(D)** On the right side of a *fgf10* mutant, the respective ventral and dorsal regions of the fused Cbs 4 and 5 are indicated by black and red arrowheads, with an arrow marking the corresponding Eb4. On the left side, five Cbs and 4 Ebs form normally. **(D’)** A high-magnification image of the right side of the *fgf10* mutant in **(D)**. Dorsal is at the bottom. **(E)** Black and red arrows indicate the respective dorsal and ventral pieces of hypoplastic Cb3 on the right side of the compound animal bearing *Tg(nkx2.3:Gal4VP16)* and *Tg(UAS:DN-EphB4a)* transgenes. On the left side, five Cbs and 4 Ebs form normally. **(E’)** A high-magnification image of the right side of the compound animal in **(E)**, with a cropped image of the dorsal piece of hypoplastic Cb3 and the corresponding Eb3 in the lower right corner. Dorsal is at the bottom. **(F)** On the right side of the compound animal transiently expressing DN-EphB4a in *nkx2.3*-positive pouch endoderm, black and red arrowheads mark the respective ventral and dorsal regions of the fused Cbs 4 and 5, with an arrow marking the corresponding Eb-like cartilage. On the left side, five Cbs and 4 Ebs form normally. **(F’)** A high-magnification image of the right side of the compound animal in **(F)**, with a cropped image of the dorsal region of the fused Cbs 4 and 5 and the corresponding Eb-like cartilage in the lower right corner. Dorsal is at the bottom. Cb, ceratobranchial cartilage; Eb, epibranchial cartilage; Pb, pharyngobranchial cartilage; AECb4, accessory element of the ceratobranchial 4; Cb1v, ventral region of Cb1; Cb1d, dorsal region of Cb1; Cb3v, ventral region of Cb3; Cb3d, dorsal region of Cb3; Cb4/5, fused Cbs 4 and 5; D, dorsal; V, ventral. n, number of animals analyzed.

Next, we genetically tested the requirement of Cbs in Eb formation. To do so, we analyzed animals with mild defects in Cbs, such as single mutants of *pax1a* and *fgf10* and transgenic animals that transiently expressed a dominant-negative form of Fgfr1 or EphB4a in *nkx2.3*-positive pouch endoderm. In these animals, one Cb was either specifically lost, hypoplastic, or fused with another Cb due to defects in the pharyngeal pouches (Choe and Crump, 2015; Jin and Choe, 2024; Jeon et al., 2025). If Eb formation depends on Cbs, then defects in Cbs would likely secondarily affect Eb formation. However, if Eb formation is independent of Cbs, similar to the independent development of the dorsal and ventral elements in the first and second arches, defects in Cbs would not necessarily influence Eb formation. Compared to wild-type siblings (Figure 4A), an 8.0 mm SL *pax1a* mutant with hypoplastic Cb2 missing its dorsal region (arrowhead in Figure 4B) showed the absence of the corresponding Eb2 (Figures 4B,B’). Consistently, in an 8.0 mm SL transgenic animal expressing a dominant-negative Fgfr1 receptor, the complete loss of left Cb4 was associated with the absence of the corresponding Eb4 (Figures 4C,C’). However, in the *pax1a* mutant with hypoplastic Cb1 bearing its separated dorsal end (black arrows in Figures 4B,B”), the corresponding hypoplastic Eb1 was observed (red arrows in Figure 4B”). Consistently, in an 8.0 mm SL transgenic animal expressing a dominant-negative form of EphrinB4a receptor with hypoplastic Cb3 bearing its separated dorsal region (black arrow in Figure 4E), the corresponding Eb3 was observed (Figures 4E,E’). In addition, in an 8.0 mm SL *fgf10* mutant with fused Cbs 4 and 5 (arrowheads in Figures 4D,D’), a corresponding Eb4 was seen at the dorsal end of the fused last ceratobranchial (arrows in Figures 4D,D’). Considering that, in 8.0 mm SL wild-type zebrafish, the dorsal area of the last Cb5 completely ossified bearing teeth, but with no alcian blue-positive cartilages, including Eb, seen (red arrowheads in Figure 4A), the alcian blue-positive cartilage in the dorsal region of the fused Cb seemed to be derived from Cb4 (red arrowheads in Figures 4D,D’), which appeared to be associated with the development of the corresponding Eb4 at the dorsal end of the last Cb (arrows in Figures 4D,D’). Similarly, in an 8.0 mm SL transgenic animal expressing a dominant-negative form of EphrinB4a receptor in which Cbs 4 and 5 were fused (arrowheads in Figures 4F,F’), hypoplastic epibranchial-like cartilage was seen at the dorsal end of the fused last Cb (red arrows in Figures 4F,F’), with alcian blue-positive cartilage visible in the dorsal region (red arrowheads in Figures 4F,F’). The correlated defects in the dorsal areas of Cb cartilages and corresponding Eb cartilages support the dependency of Eb formation on the dorsal end of Cb. However, we still could not completely rule out the other possibility that independent populations of chondrocytes for Ebs are present, but so close to the dorsal ends of the corresponding Cbs that the loss of the dorsal ends of Cbs simultaneously accompanies the loss of the independent chondrocyte populations of Ebs.

### Fgf3 may be required for epibranchial cartilage formation

**FIGURE 5:**
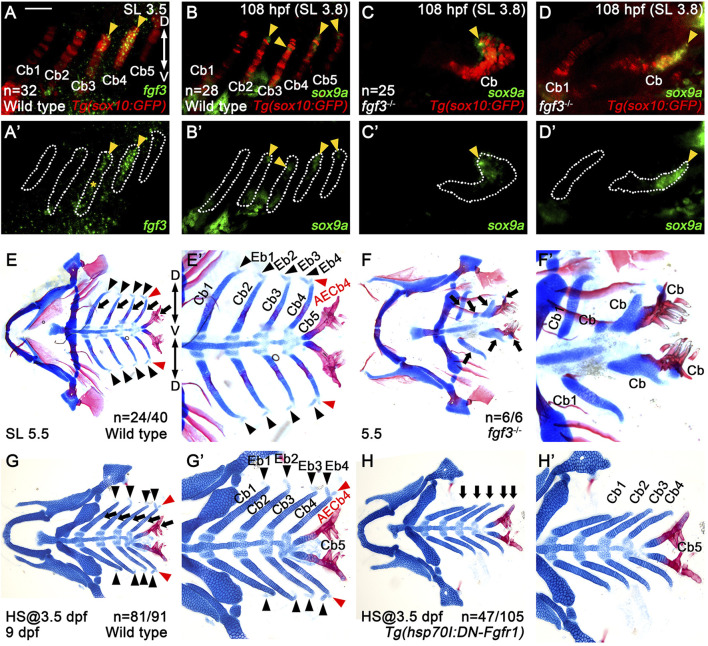
Requirement of Fgf3 in epibranchial cartilage development. **(A–D)** Fluorescence *in situ* hybridization of *fgf3* (green) or *sox9a* (green) in conjunction with the GFP immunohistochemistry (red) in *Tg(sox10:GFP)* reporter lines. **(A’–D’)** Green channel only with the Cbs outlined. Scale bar: 40 μm. Anterior is to the left. Dorsal is at the top. **(A,A’)** *fgf3* expression in 3.5 mm SL wild-type zebrafish. Arrowheads indicate *fgf3* expression in the middle and dorsal regions of Cb4 and the dorsal region of Cb3. **(A’)** * marks *fgf3* expression in the adjacent regions to the ventral area of Cb3. **(B–D)** *sox9a* expression in wild types **(B)** and *fgf3* mutants **(C,D)** at 108 hpf and 3.8 mm SL. Arrowheads mark *sox9a* expression in the dorsal areas of Cbs 2-5 in wild types **(B)** and at the ends of distorted Cbs in severe **(C)** and intermediate **(D)** *fgf3* mutants. In wild types, *sox9a* expression is not seen in Cb1 **(B)**. In severe *fgf3* mutants, Cb1 is missing **(C)**. In intermediate *fgf3* mutants, Cb1 forms, but with no *sox9a* expression seen **(D)**. **(E–H)** Craniofacial skeletal elements stained with Alcian Blue (cartilage) and Alizarin Red (bone). Arrows indicate Cbs, with black arrowheads marking Ebs. AECb4 is marked with red arrowheads. **(E’–H’)** Cropped images of the branchial basket corresponding to **(E–H)**. **(E,E’)** In 24 out of 40 wild-type siblings, 4 Ebs on each side bud off at the dorsal end of the corresponding Cbs, along with AECb4 appearing on the posterior to Eb4 at 5.5 mm SL. The remaining 16 animals form 3 Ebs on each side, yet no Eb1. **(F,F′)** In all 6 5.5 mm SL *fgf3* mutant siblings, Cb1s are relatively normal, with the other Cbs being fused or missing. Eb1 is not observed at the dorsal end of the corresponding Cb1, with no Ebs seen at the dorsal end of fused Cbs. **(G,G’)** In 81 out of 91 wild-type siblings treated with heat shock at 3.5 dpf for 3 days, 4 Ebs on each side form at the dorsal end of the corresponding Cbs at 9 dpf. The remaining 10 animals form 3 Ebs on each side, yet no Eb1. All 91 animals develop 5 Cbs with normal shapes. **(H,H’)** In 47 out of 105 *Tg(hsp70I:DN-Fgfr1)* animals treated with heat shock at 3.5 dpf for 3 days, no Ebs form at the dorsal end of the corresponding Cbs at 9 dpf. 44 animals form 2 Ebs (Ebs 1 and 2), without Ebs 3 and 4. The remaining 14 fish display 3 Ebs (Ebs 1-3 or Ebs 1, 3, and 4) but no Eb 2 or 4. In all 105 fish, 5 Cbs with normal shapes form. Cb, ceratobranchial cartilage; Eb, epibranchial cartilage; AECb4, accessory element of the ceratobranchial 4; D, dorsal; V, ventral. Anterior is to the left. n, number of animals analyzed.

Given the importance of pharyngeal pouches in controlling the development of Cbs required for Eb formation (Piotrowski and Nüsslein-Volhard, 2000), we examined the genes necessary for developing pharyngeal pouches to potentially identify those involved in Eb formation. As single mutants for *tbx1*, *fgf3*, and *fgf8a*, and double mutants for *pax1a* and *pax1b* display severe defects in pouches and Cbs in zebrafish (Piotrowski et al., 2003; Crump et al., 2004a; Herzog et al., 2004; Liu et al., 2020), we screened these genes in Eb formation. Interestingly, among them, we found *fgf3* expression in the developing branchial basket. At 3.5 mm SL, before Eb4 appeared at the dorsal end of Cb4, we observed *fgf3* expression in the dorsal and intermediate regions of Cb4, with a relatively mild expression of *fgf3* in the dorsal area of Cb3, implying a potential role of Fgf3 in Eb formation (arrowheads in Figures 5A,A’).

To investigate the role of Fgf3 in Eb formation, we established the *fgf3* ^*GNU48*^ mutant line using the CRISPR/Cas9 system (Supplementary Figures S2A,B). Although most of the *fgf3* ^*GNU48*^ mutants did not survive beyond 16 dpf or grow more than 4.0 mm SL (Supplementary Figures S2C,D), we found a very small number of *fgf3* ^*GNU48*^ mutants that survived up to 26 dpf and grew to 5.5 mm SL. This allowed us to analyze Eb formation in the *fgf3* ^*GNU48*^ mutants. In the mutants, most Cbs were fused or missing, but the first Cbs were relatively normal (arrows in Figures 5F,F’). Interestingly, compared to 5.5 mm SL wild types in which all 4 Ebs formed on each side in 24 out of 40 animals (black arrowheads in Figures 5E,E’), all six 5.5 mm SL *fgf3* mutants did not form any Ebs at the dorsal ends of Cbs, even in relatively normal Cb1 (Figures 5F,F’).

However, the absence of Ebs at the dorsal ends of normal and fused Cbs in the *fgf3* mutants could be the secondary consequence of the loss of the dorsal regions of the Cbs, given the importance of Fgf3 in the development of pouches and, subsequently, Cbs. To investigate this possibility, we examined whether the distorted Cbs of the *fgf3* mutants still had a dorsal region by analyzing *sox9a* expression. In wild types, at 108 hpf, the *sox9a* expression in the posterior arches 4-7 was localized to the dorsal areas, with its expression in arch 3 abolished (Figure 2G). We verified that the localized domains of *sox9a* expression at 108 hpf were the dorsal regions of Cbs 2–5 (arrowheads in Figures 5B,B’). In the *fgf3* mutants, *sox9a* expression was still observed at the ends of the distorted Cbs, except for the relatively normal Cb1, at 108 hpf (arrowheads in Figures 5C,D). This result suggests that the distorted Cbs still maintain the *sox9a*-positive dorsal regions in the *fgf3* mutants. Thus, the absence of Ebs observed in *fgf3* mutants was not likely due to the secondary consequence of the loss of dorsal regions of Cbs. Based on the expression and functional analyses of *fgf3* in Eb formation, we propose a potential role of Fgf3 in developing Eb cartilages.

In order to further verify the role of Fgf in Eb formation, we prevented Fgf signaling specifically during Eb formation with *Tg(hsp70I:DN-Fgfr1)* transgene after Cb development (Jeon et al., 2025). Heat shock treatment in *Tg(hsp70I:DN-Fgfr1)* transgenic fish from 3.5 to 6.5 dpf resulted in the absence of all Ebs but with Cbs unaffected (Figures 5H,H’) at 9 dpf, compared to wild-type siblings treated with heat-shocking in which Ebs and Cbs formed normally (Figures 5G,G’). This result implies that Fgf signal is necessary for Eb formation and further supports the role of Fgf3 in Eb formation.

### Fgf3 may control the proliferation of cartilage cells in the dorsal area of ceratobranchial cartilage in epibranchial cartilage formation

**FIGURE 6:**
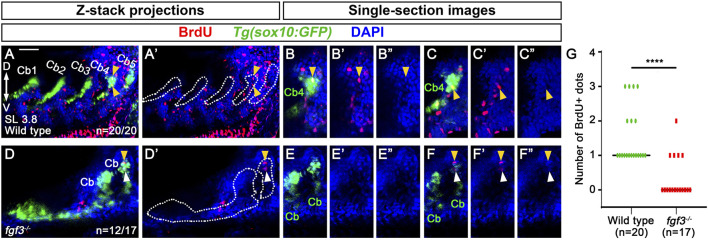
Requirement of *fgf3* in proliferating chondrocytes in the dorsal area of ceratobranchial cartilage 4 in epibranchial cartilage 4 formation. **(A–F)** BrdU (red) and DAPI (blue) staining relative to *sox10*-positive Cbs (green) in 3.8 mm SL wild-type siblings and *fgf3* mutants. Cbs are outlined in **(A’,D’)**. **(A’–F’)** Red and blue channels only. **(B”, C”, E”, F”)** Blue channel only. **(A,A’)** Confocal z-stack projections showing BrdU-positive dots in wild types. All 20 animals show BrdU-positive dots in GFP-positive cells of the dorsal area of Cb4 (arrowheads). **(B–C”)** Confocal single-section images isolated from **(A)** that focus on the dorsal area of Cb4. Arrowheads indicate BrdU-positive dots colocalizing with DAPI and GFP in the dorsal region of Cb4. **(D,D′)** Confocal z-stack projections showing BrdU-positive dots in *fgf3* mutants. 12 out of 17 animals do not show BrdU-positive dots in GFP-positive cells of the dorsal areas of distorted Cbs, with 4 of them showing BrdU-positive dots in GFP-negative cells of the adjacent regions to the dorsal areas of distorted Cbs (arrowheads). The remaining 5 animals display BrdU-positive dots in GFP-positive cells of the dorsal regions of the distorted Cbs. **(E–E”)** A confocal single-section image isolated from **(D)** that focuses on the dorsal area of the distorted Cb. No BrdU-positive dots are observed in the GFP-positive dorsal regions of the distorted Cbs. **(F–F”)** A confocal single-section image isolated from **(D)** that focuses on BrdU staining in the adjacent region of the dorsal area of the distorted Cb. Yellow arrowhead indicates a BrdU-positive dot colocalizing with DAPI but not with GFP in the adjacent dorsal region of the distorted Cb. White arrowhead marks BrdU-positive staining that does not colocalize with DAPI and GFP in the adjacent dorsal region of the distorted Cb. **(G)** Quantification of the number of BrdU-positive dots colocalizing with DAPI and GFP in the dorsal region of Cbs analyzed in **(A,D)**. Data is represented on a scatter plot. **** shows p < 0.0001. Cb, ceratobranchial cartilage; D, dorsal; V, ventral. Anterior is to the left. Dorsal is at the top. Scale bar: 40 μm. n, number of animals analyzed.

Next, we investigated the cellular requirements of Fgf3 in Eb formation. Since we observed the stratification of cartilage cells in the dorsal region of Cb4, we first examined cell proliferation using BrdU staining in 3.8 mm SL *Tg(sox10:GFP)* zebrafish labeling Cbs. When Eb4 was about to form at 3.8 mm SL, BrdU-positive dots were seen in the nuclei of GFP-positive cells in the dorsal region of Cb4 in all 20 animals (arrowheads in Figures 6A–C”). However, BrdU-positive dots were rarely observed at the dorsal ends of Cbs 1–3 (Figures 6A,A’). This result suggests that cell proliferation in the dorsal end of Cb4 may play a role in the stratification of cartilage cells in Eb4 formation.

Given the observed proliferation of GFP-positive cartilage cells in the dorsal area of Cb4 in wild types, we analyzed whether Fgf3 was involved in the process. In 12 out of 17 3.8 mm SL *fgf3* mutants, BrdU-positive cells were not seen in the GFP-positive cells in the dorsal regions of the distorted Cbs. However, in 4 out of the 12 *fgf3* mutants, one or two BrdU-positive dots were seen in the adjacent GFP-negative cells to the dorsal areas of the distorted Cbs (arrowheads in Figures 6D–F”). The remaining 5 *fgf3* mutants showed BrdU-positive dots in the dorsal areas of the distorted Cbs as wild types. Compared to wild types, the reduced cell proliferation in the dorsal regions of Cbs in *fgf3* mutants was statistically significant (Figure 6G). To verify further the role of Fgf3 in the proliferation of cartilage cells in Eb formation, we tried to analyze MAPK (ERK1/2) signaling in 3.8 mm SL zebrafish, as Fgf-induced cell proliferation is dependent on MAPK (ERK1/2) signaling (Lovicu and McAvoy, 2001; Goldshmit et al., 2012). However, due to technical issues, our whole-mount immunohistochemistry of phospho-ERK1/2 in 3.8 mm SL zebrafish larvae did not work. Nonetheless, the reduced cell proliferation in the dorsal regions of Cbs in *fgf3* mutants suggests that Fgf3 may control the local proliferation of cartilage cells in the dorsal area of Cbs in Eb formation.

## Discussion

In this study, we report the development of Ebs arising in the posterior pharyngeal arches in zebrafish, which shows several features previously unappreciated in other fish. First, Eb cartilages form sequentially in a posterior-to-anterior progression. Second, Eb cartilages form from chondrocytes at the dorsal end of Cb cartilage. Third, Fgf3 is necessary for Eb cartilage formation, possibly by regulating the proliferation of chondrocytes in the dorsal region of Cb cartilage, which would promote stratification. Our results provide a novel insight into the development of Ebs and establish a genetic and cellular basis to investigate their developmental mechanism.

### Epibranchial cartilage formation may probably follow branching morphogenesis

**FIGURE 7:**
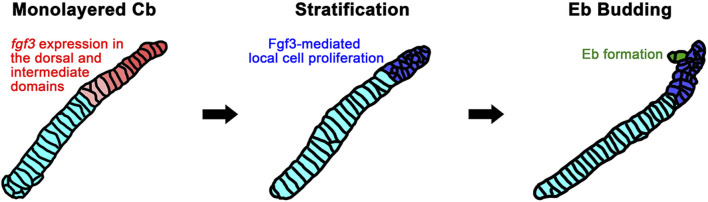
A model for Fgf3-dependent epibranchial formation. The expression of *fgf3* appears in the dorsal and intermediate domains of monolayered ceratobranchial cartilage before epibranchial cartilage formation begins. This *fgf3* expression would promote the proliferation of chondrocytes in the dorsal area of ceratobranchial cartilage to lead to stratification. Some of the chondrocytes from the stratified dorsal end would migrate to form an epibranchial bud. Cb, ceratobranchial cartilage; Eb, epibranchial cartilage.

Although it has been reported in fish that Eb cartilage arises at the dorsal end of corresponding Cb cartilage as a separate chondrification center (Carvalho et al., 2013), we observed that zebrafish Eb cartilage begins to form by budding out chondrocytes at the dorsal end of corresponding Cb cartilage (Figure 7). Then, the bud seems to grow by cell rearrangement to extend Eb cartilage. This developmental process is almost identical to that described in the branching morphogenesis of pharyngeal pouches (Choe et al., 2013). In both, the mono-layered cells stratify into the multi-layered cells, and then a few cells migrate out to form a bud. The bud grows by cell rearrangement. In Eb formation, the stratification occurs in the dorsal area of Cb cartilage, probably through a local proliferation of chondrocytes upon receiving Fgf3 signal (Figure 7), as in the stratification of pharyngeal endoderm by Wnt11r signal to form pouches (Choe and Crump, 2014). It would also be feasible that the growth zone at the dorsal end of Cb cartilage, previously identified, could simultaneously provide chondrocytes for branching Eb cartilage as well as for the growth of Cb cartilage itself (Heubel et al., 2021; Le Pabic et al., 2022).

How the chondrocytes bud out anterior-dorsally in Eb cartilage formation remains to be determined. One immediate expectation would be the presence of a guidance cue, as in the guided migration of pouch-forming cells by the mesodermal Fgf8a signal in the branching morphogenesis of pouches (Choe and Crump, 2014). However, in our preliminary analysis of *fgf8a* mutants, Eb buds seemed to form, implying that Fgf8a might not play a similar guidance role in Eb formation. Alternatively, oriented cell division could contribute to bud out chondrocytes anterior-dorsally for Eb cartilage formation, similar to that described in the branching morphogenesis of zebrafish vessels and mouse lungs (Zeng et al., 2007; Tang et al., 2011).

### Chondrocytes of ceratobranchial cartilages would establish the dorso-ventral identity that subsequently influences the development of epibranchial cartilages

The dependency of Eb cartilage formation on the dorsal region of Cb cartilage implies that the dorso-ventral identity of chondrocytes in the simple rod-shaped Cb cartilage might be established. Consistently, we observed *fgf3* and *sox9a* expression in the dorsal domains of Cb cartilages. While the dorso-ventral patterning in the pharyngeal arches by the Dlx code is well-understood (Talbot et al., 2010; Gillis et al., 2013), it has yet to be investigated how chondrocytes of Cb cartilages establish the dorso-ventral identity during the morphogenesis of Cb cartilages. Considering the ventral domain of arches is neighbored interiorly by the paraxial mesoderm-derived core and exteriorly by pharyngeal ectoderm, those required for ventral cartilage formation (Yelick and Schilling, 2002), these tissues could contribute to establishing the dorso-ventral identity of chondrocytes in Cb cartilages, in addition to their contribution to the early dorso-ventral patterning of the arches (Clouthier et al., 2000; Miller et al., 2000). It would also be possible that pharyngeal pouches that segment the pharyngeal arches along the dorso-ventral axis provide localized signals for the adjacent chondrocytes to acquire the dorso-ventral identity in the morphogenesis of Cb cartilages. Indeed, in hyosymplectic cartilage development, we found a late role of the first pouch in the morphogenesis of the dorsal domain of hyosymplectic cartilage through the Pax1a-EphrinB2a pathway (Jeon et al., 2025), in addition to the previously reported early role in segmenting the dorsal region of the second arch (Crump et al., 2004b). Identifying molecular markers labeling the dorsal and ventral chondrocytes in ceratobranchial cartilages, in addition to *fgf3* and *sox9a*, which are expressed in the dorsal domain of Cb cartilages, and understanding the regulation of these markers will provide better insight into the dorso-ventral identity of ceratobranchial cartilages.

### Epibranchials may not be serially homologous to the dorsal skeletal elements of the first and second arches

Generally, it is recognized that the ventral Cb cartilages are serially homologous to the Meckel’s and ceratohyal cartilages, and the dorsal Eb cartilages are serially homologous to the palatoquadrate and hyosymplectic cartilages (Schilling and Kimmel, 1997). While the homology of the palatoquadrate and hyosymplectic cartilages is supported by similar dorsal positions and shapes, as well as by the developmental genetic study of their homologs in mice, zebrafish, and skate, the homology of Eb cartilages that are smaller and shaped differently than the palatoquadrate and hyosymplectic cartilages has yet to be determined (Rijli et al., 1993; Schilling and Kimmel, 1997; Talbot et al., 2010; Gillis et al., 2013). Our observation gives rise to a suspicion of their supposed serial homology to the dorsal skeletal elements of the first and second arches in that Eb cartilages originate in the same skeletogenic mesenchymal condensations to Cb cartilages rather than in separate condensations in the posterior pharyngeal arches, as well as by budding out chondrocytes in the dorsal area of Cb cartilages. However, our understanding of the development of facial skeletal elements in the posterior arches is too restricted to test our suspicion compared to the development of facial skeletal elements in the first and second arches. Detailed genetic and cellular analysis in the development of Cb and Eb cartilages will provide better insight into the serial homology of these skeletal elements in the posterior arches to those in the first and second arches.

## Data Availability

The original contributions presented in the study are included in the article/Supplementary Material, further inquiries can be directed to the corresponding author.
