# Supplementary material for: A potential role of Fgf3 for epibranchial formation in zebrafish
Source: Front Cell Dev Biol. 2025 Aug 20;13:1652723. doi: 10.3389/fcell.2025.1652723 (PMC12405170; doi:10.3389/fcell.2025.1652723)
Supplement: Supplementary file 1 [file DataSheet1.pdf]

SUPPLEMENTARY MATERIALS AND METHODS

gRNA oligo used to generate loss of function *fgf3* gene

fgf3:

5'-GCGTAATACGACTCACTATAGGGGCCAGGCGTGTGACCCAGTTTTAGAGCTAGAAATAGCAAGTTAAAATAAGGCTAGTCCGTTATCAACTTGAAAAAGTGGCACCGAGTCGGTGCTTT-3'

Genotyping primers

*fgf3*_GT_F: 5'-CGGCTAACTGGATCACTG-3'

*fgf3*_GT_R: 5'-CCGTCTATTTTCCCGTTC-3'

Primers used to create in situ probes

hand2-r-F: 5'-ACCATGACGGCTATTCCT-3'

hand2-r-R: 5'-ATTGCTGCTCCCTGAACT-3'

jag1b-r-F: 5'- ACGTGACGAGTTCTTTGG-3'

jag1b -r-R: 5'-GGCATGACTTAGCGTTCA-3'

dlx2a-r-F: 5'-AACAGCGTCCAGTACAAC-3'

dlx2a-r-R: 5'-AGAAAGGAAGGGGTGGTT-3'

dlx3b-r-F: 5'-CAGAGCCCTCCATATCCT-3'

dlx3b-r-R: 5'-AGAGTGCGCGTTATTGTC-3'

dlx4a-r-F: 5'-TCAAAGTCGGCTTTTCTG-3'

dlx4a-r-R: 5'-TGTTCTCCTTCAGGTCCA-3'

barx1-r-F: 5'-AGTTTCCGGTGTCTCCTC-3'

barx1-r-R: 5'-CCTCTTGGTTTGCATCAG-3'

sox9a-r-F: 5'-AGCAGCGATGTTATCGAA-3'

sox9a -r-R: 5'-GGACTGTGGTTGGATTGA-3'

SUPPLEMENTARY FIGURES

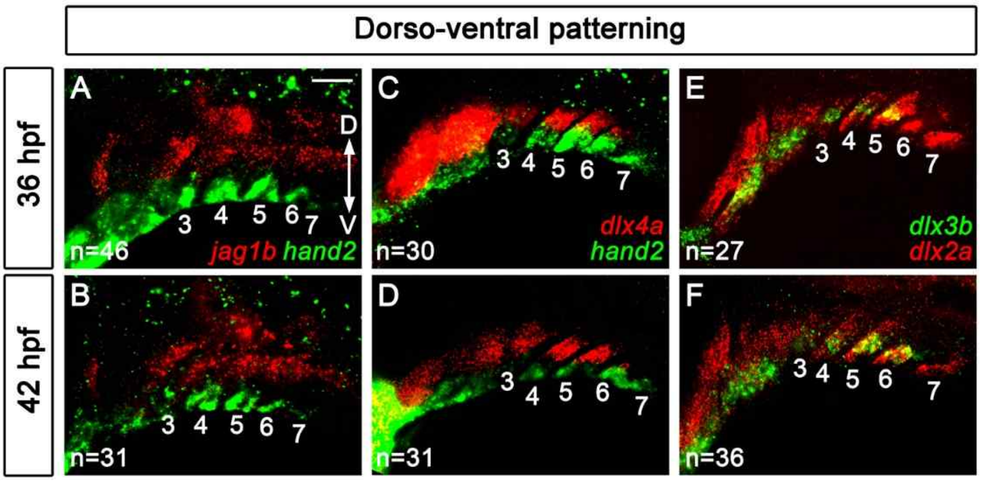

Supplementary Figure 1. Dorso-ventral patterning in the posterior arches.

(A-F) Double fluorescence in situ hybridization of *jag1b* (red) and *hand2* (green), *dlx4a* (red) and *hand2* (green), and *dlx2a* (red) and *dlx3b* (green) at 36 and 42 hpf. Posterior arches are numbered.

(A-D) Expression of *hand2* is detected in the ventral areas of arches 3 to 7 at 36 hpf (A and C) and is maintained at 42 hpf (B and D). At 36 and 42 hpf, *jag1b* expression is observed in the dorsal domain of arches 3 to 7 (A and B). *dlx4a* expression is seen in the intermediate domain of arches 3 to 6 at 36 (C) and 42 hpf (D), with no expression observed in arch 7, the last arch, until 42 hpf.

(E, F) Expression domain of *dlx2a* marks each pharyngeal arch at 36 and 42 hpf. *dlx3b* is expressed in the intermediate domain of arches 3 to 5 at 36 hpf (E). At 42 hpf, *dlx3b* expression is seen in the intermediate domain of arches 3 to 6, but with no expression in the last arch 7 (F).

Scale bar: 40 µm. Anterior is to the left. Dorsal is at the top.

D, dorsal; V, ventral.

n, number of animals analyzed.

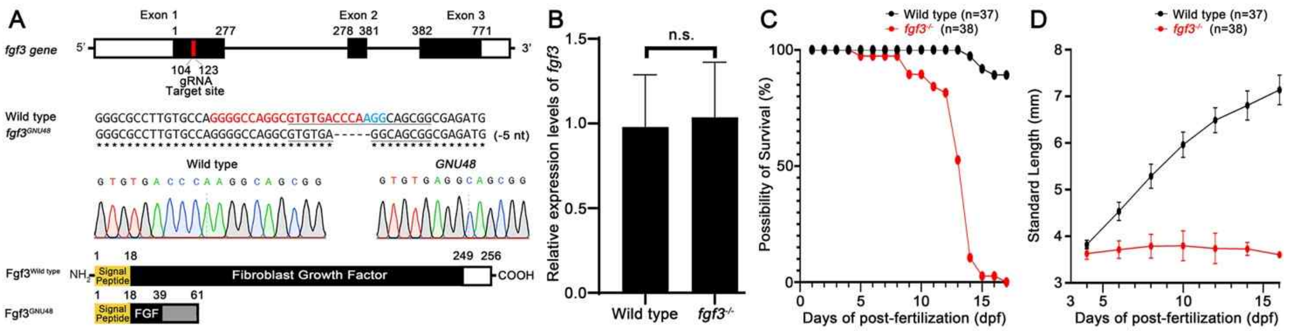

Supplementary Figure 2. Generation of a loss-of-function allele of the *fgf3* gene. **(A)** The *fgf3* gene contains three exons bearing sequences for the protein-coding region (black box) and the 5’ and 3’ untranslated regions (open box). The gRNA target site is marked in red. The deletion mutation of the mutant allele is shown in the multiple sequence alignments, with the gRNA target and the PAM sites being marked in red and blue, respectively, in the wild-type sequence. The electrophoretograms show the lesion in the mutant allele underlined in the multiple sequence alignments. Schematics of the Fgf3 proteins encoded by the wild-type and mutant alleles show early truncation in the mutant Fgf3 protein, with an extra non-specific region (grey box). The signal peptide is marked with a yellow box.

(B) Relative expression levels of *fgf3* mRNAs in wild types and *fgf3* mutants. Expression in wild types set at 1. Data is represented on a column bar graph. n.s., not significant.

(C) Survival plots of wild-type siblings and *fgf3* mutants.

(D) Growth plots of wild-type siblings and *fgf3* mutants.
